# Supplementary material for: Domain-dependent effects of insulin and IGF-1 receptors on signalling and gene expression
Source: Nat Commun. 2017 Mar 27;8:14892. doi: 10.1038/ncomms14892 (PMC5378997; doi:10.1038/ncomms14892)
Supplement: Supplementary Information — Supplementary Figures, Supplementary Tables. [file ncomms14892-s1.pdf]

Supplementary Figure 1

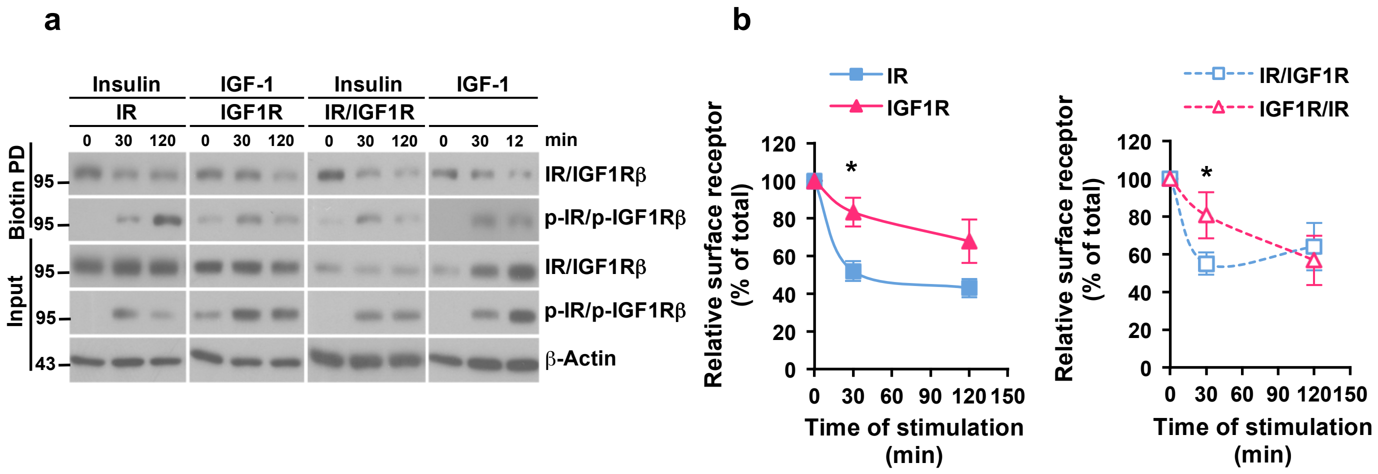

Supplementary Figure 1. Receptor internalization following ligand binding. (a) Immunoblotting of total and phosphorylated IR and IGF1R beta-subunit in cell lysates and biotin-labeled cell surface fraction. Biotinylated fractions of the receptors pulled-down (PD) by Streptavidin Agarose beads are shown on the top. Receptor expression from the total lysates are shown at the bottom. **(b)** Quantification of relative surface receptor amount at different time point following initial administration of insulin or IGF-1. Data are presented as mean ± SEM (*, $p < 0.05$; two-way ANOVA followed by Bonferroni *post hoc* tests, n = 3).

Supplementary Figure 2

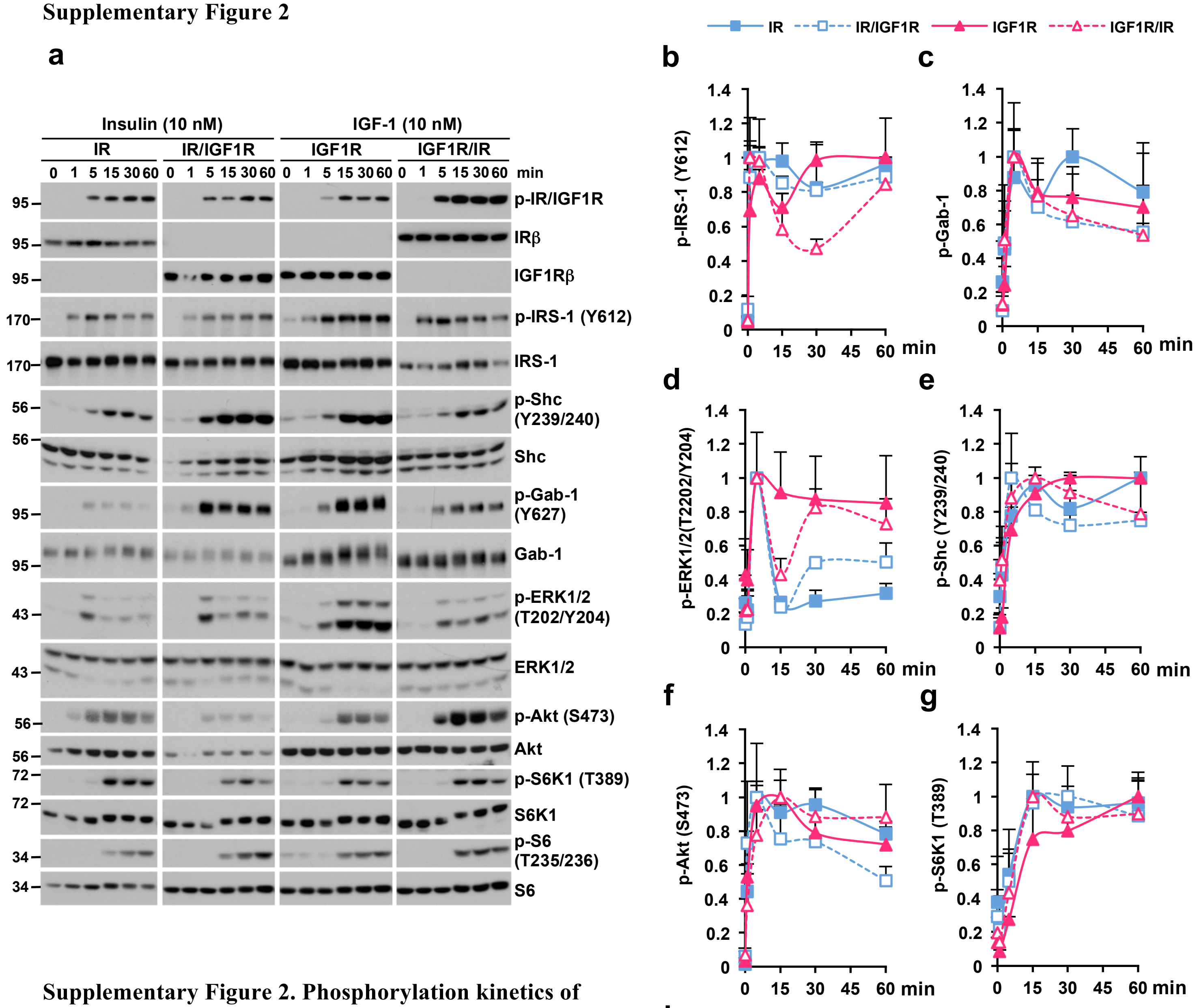

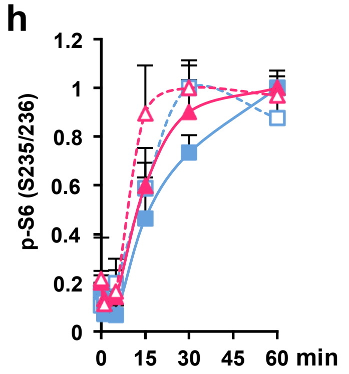

Supplementary Figure 2. Phosphorylation kinetics of common intracellular signaling targets of IR and IGF1R. **(a)** Immunoblot analysis of protein phosphorylation following 10 nM insulin or IGF-1 stimulation for the indicated time duration. **(b-h)** Densitometry analysis of relative phosphorylation of each protein along the time-course. Data was normalized to maximal phosphorylation. Data is presented as mean + SEM. n = 3.

Supplementary Figure 3

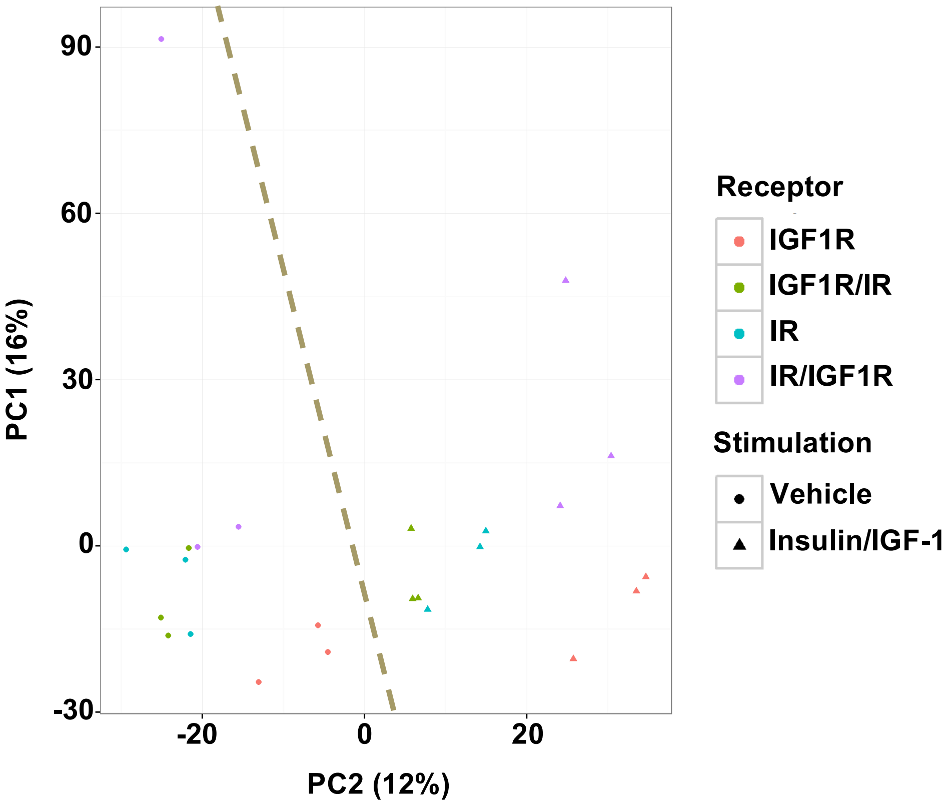

Supplementary Figure 3. PCA plots of cells expressing 4 types of receptors in response to ligand stimulation. Each dot represents one cell line under one experimental condition (with or without ligand stimulation). At basal conditions (round dots), all four cell lines segregated on one principle component (PC1). Despite some degrees of clonal variation within each type of cell, the cells expressing receptors with intracellular domain of IR (IR and IGF1R/IR) tended to cluster together, whereas cells expressing normal IGF1R segregate further away from all other cell types. Upon ligand stimulation, all four types of cells showed a dramatic stimulation effect with segregations on the other principle component (PC2). Note, one clone of IR/IGF1R cells was an extreme outlier and excluded from the following bioinformatic analysis. See details in *Methods*.

Supplementary Figure 4

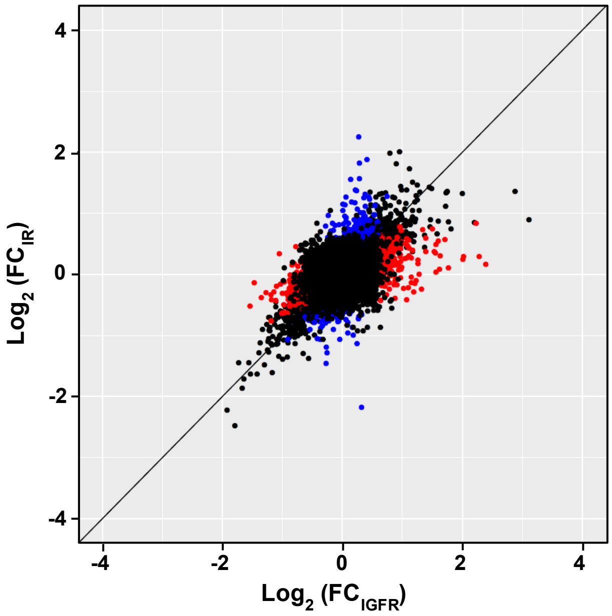

Supplementary Figure 4. Similar overall gene expression regulation of insulin on IR and IGF-1 on IGF1R. Dot plot showing distribution of fold change for gene expression after stimulation of cells expressing IR (*x*-axis) versus that in IGF1R (*y*-axis). Note the majority of the genes fall along the diagonal, indicating similar regulation by IR and IGF1R in response to insulin and IGF-1 stimulation, respectively. Blue dots represent the genes specifically regulated by IR by at least 50% (FDR < 0.05), but not IGF1R (FDR ≥ 0.05); red dots represent the genes specifically regulated by IGF1R by at least 50% (FDR < 0.05), but not IR (FDR ≥ 0.05).

Supplementary Figure 5

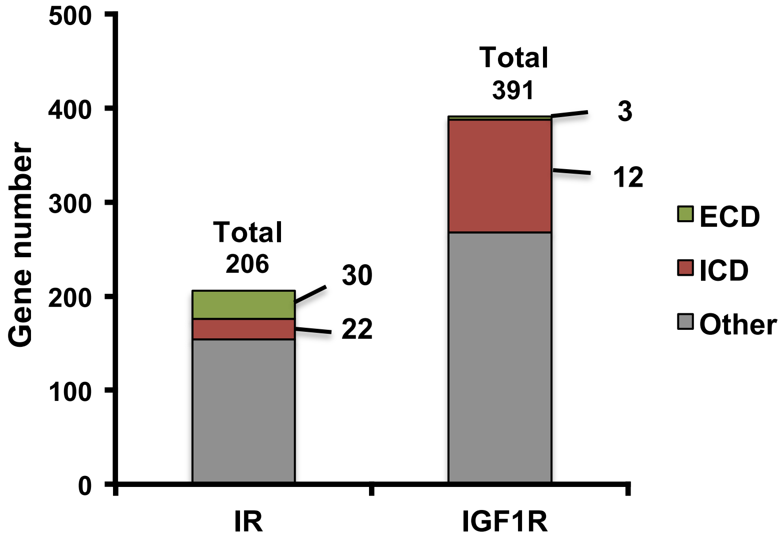

Supplementary Figure 5. Representation of genes uniquely regulated by IR and IGF1R corresponding to the intracellular and extracellular domains of the receptors. Among the 206 genes that were uniquely regulated by IR, 30 (15%) corresponded to the extracellular domain of the IR, whereas 22 (11%) corresponded to the intracellular domain of the IR. In contrast, of the 391 genes that were only regulated by IGF1R, only 3 (~1%) corresponded to the extracellular domain of the IGF1R, while 120 (31%) corresponded to the intracellular domain of the IGF1R.

Supplementary Figure 6

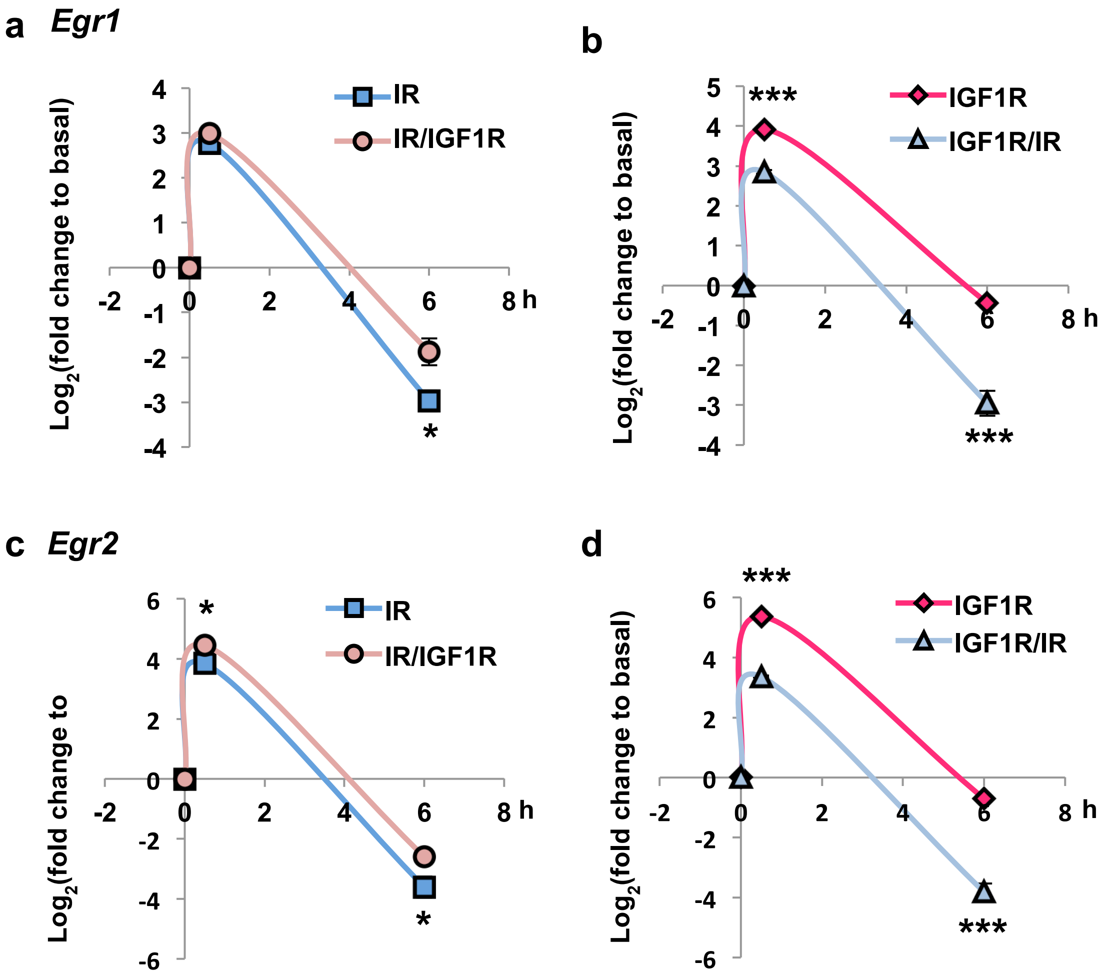

Supplementary Figure 6. Time course of *Egr1* and *Egr2* gene expression. Fold change of *Egr1* (a and b) and *Egr2* (c and d) on a $\log_2$ scale in response to 100 nM insulin or 100 nM IGF-1 stimulation for 30 min and 6 hours were confirmed by qPCR, with TBP as the housekeeping gene. Data are presented as mean ± SEM (*, $p < 0.05$; *** $p < 0.001$. Student *t*-test. n = 6).

Supplementary Figure 7

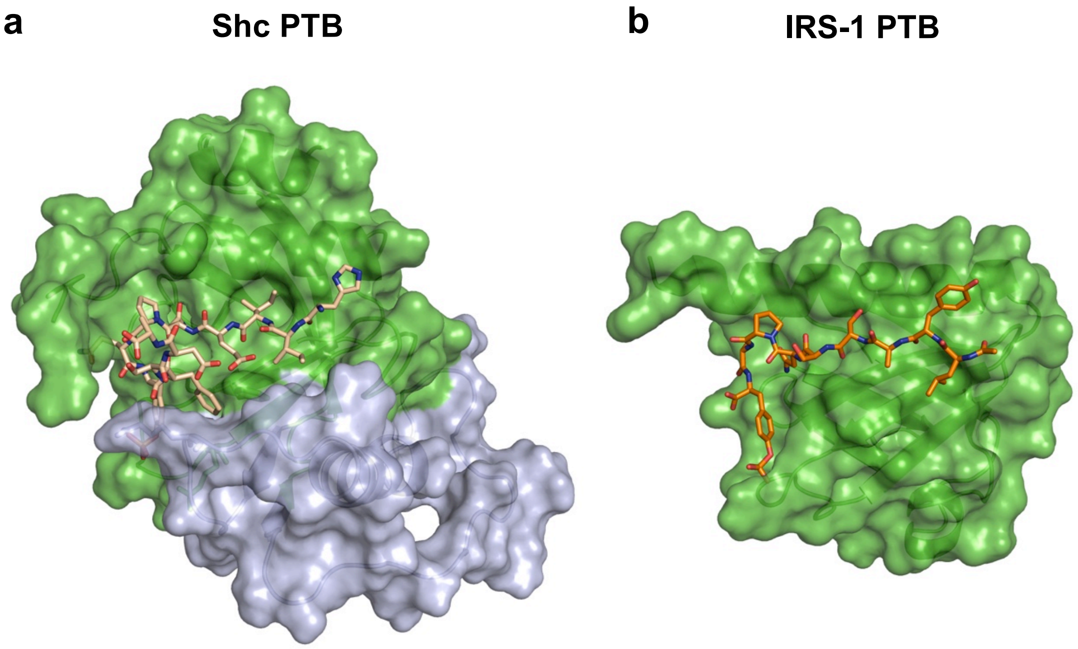

Supplementary Figure 7. Surface representations of the Shc PTB and IRS-1 PTB domains. (a) Surface representation of the solution NMR structure of the Shc PTB domain (green and grey) bound to a phosphorylated segment containing the NPxY motif from the TrkA receptor (ribbon). Structure modelled from Protein Data Bank deposition 1SHC. **(b)** Surface representation of the crystal structure of the IRS-1 PTB domain (green) bound to a phosphorylated segment of the juxtamembrane region of the insulin receptor (ribbon). Structure modelled from Protein Data Bank deposition 5U1M.

Supplementary Figure 8

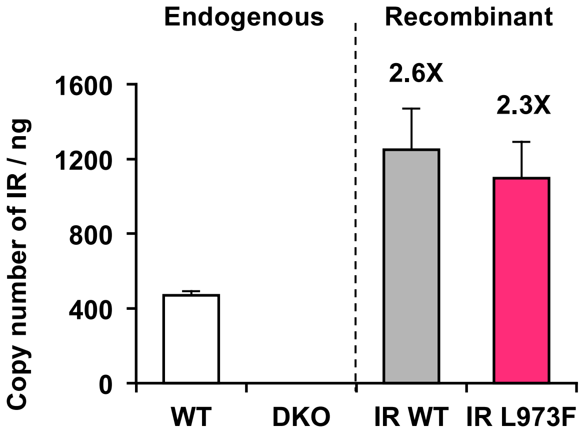

Supplementary Figure 8. Expression levels of receptors in cells expressing IR and IR^{L973F}. Relative mRNA levels of endogenous IR from wild-type and DKO cells, as well as over-expressed recombinant receptors, wild-type human IR and IR^{L973F}. Data are shown as mean ± SEM copy number per ng total RNA. n = 3-5. Fold over-expression of each recombinant receptor to endogenous receptor is indicated.

Supplementary Figure 9

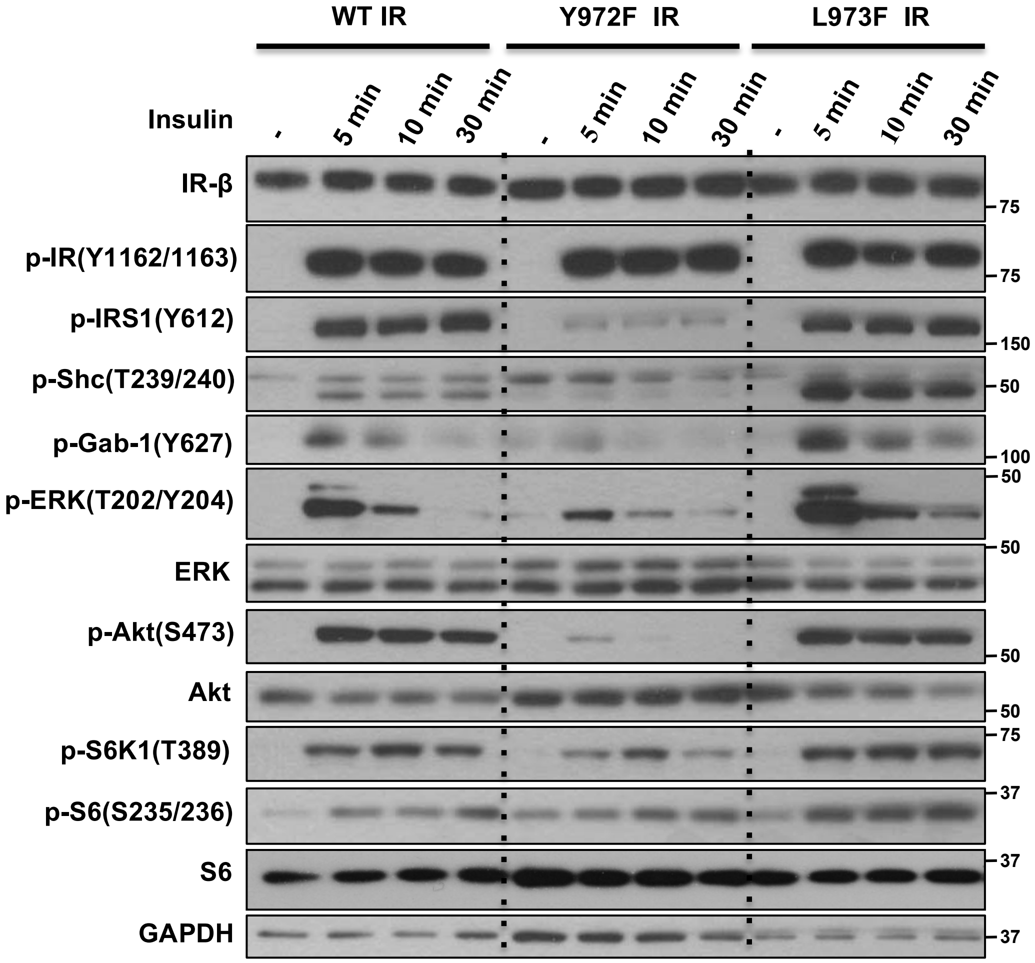

Supplementary Figure 9. Phosphorylation kinetics of common intracellular signaling targets of IR, IR Y972F and IR L973F. Immunoblot of protein phosphorylation following insulin stimulation for indicated time duration and antibodies.

Supplementary Figure 10

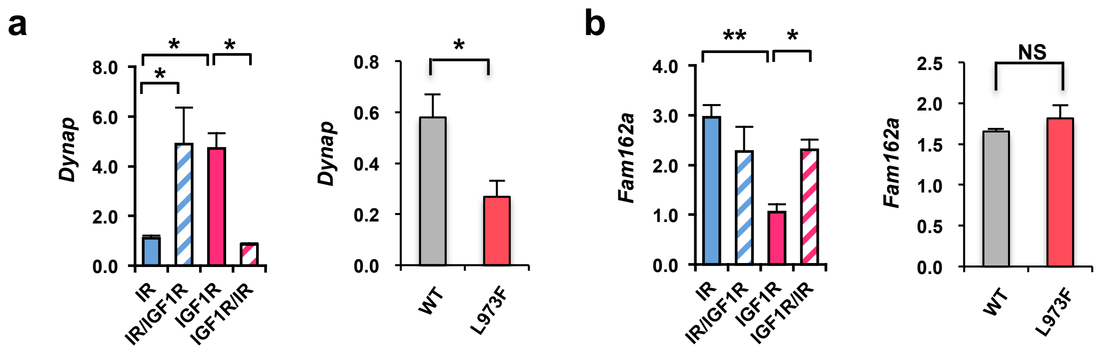

Supplementary Figure 10. Gene expression dependent on ICD of IR and IGF1R receptors but not dependent on Leu^{973} in IR and Phe^{951} in IGF1R. (a) Fold changes of expression in response to stimulation for *Dynap*, which is highly regulated by IGF1R-ICD, were confirmed in both chimeric receptor and point mutated IR receptor systems by qPCR, with TBP as the housekeeping gene. **(b)** Fold changes of expression in response to stimulation for *Fam162a*, which is highly regulated by IR-ICD were confirmed in both chimeric receptor and point mutated IR receptor systems by qPCR, with TBP as the housekeeping gene. Data are presented as the mean ± SEM (*, $p < 0.05$; **, $p < 0.01$; *** $p < 0.001$. One-way ANOVA followed by Newman-Keuls *post-hoc* analysis or Student *t*-test when appropriate, n = 3-6).

Supplementary Figure 11

Figure 1c

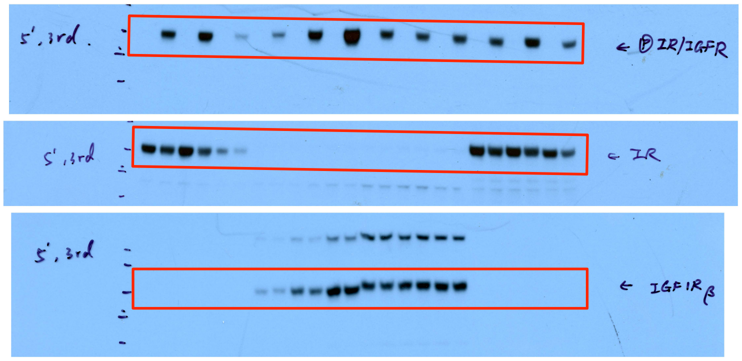

Figure 2a

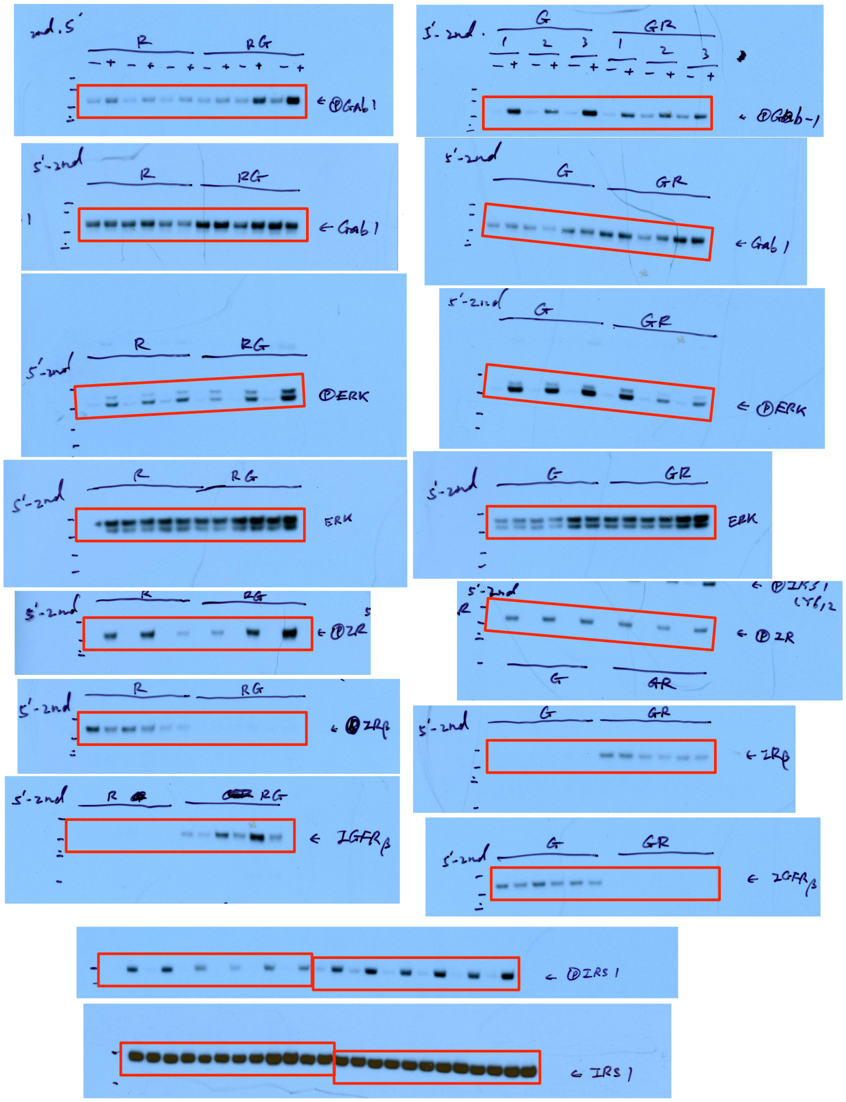

Supplementary Figure 11. Uncut blots. The red sections indicate blot results shown in the indicated figures.

Supplementary Figure 12

Figure 2e

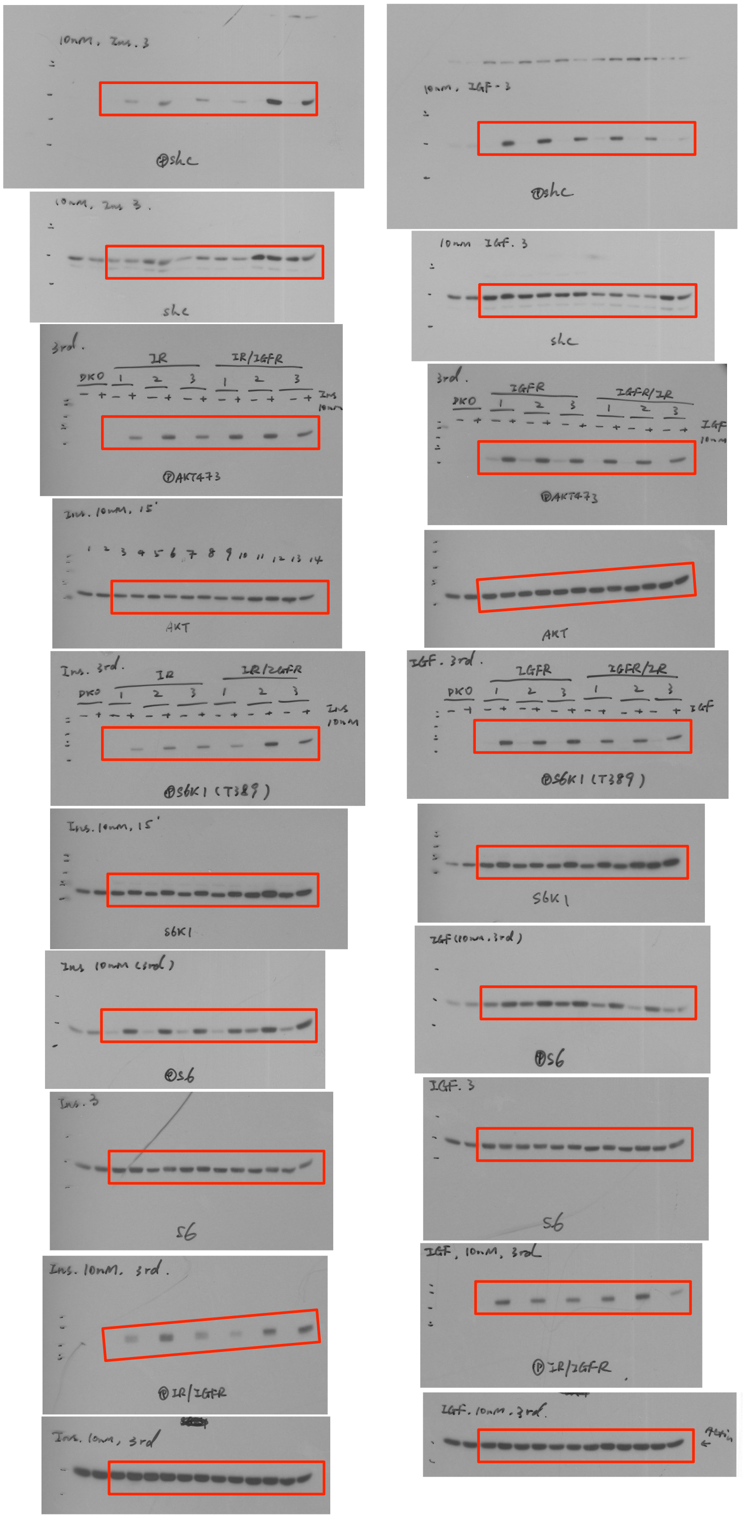

Supplementary Figure 12. Uncut blots. The red sections indicate blot results shown in the indicated figures.

Supplementary Figure 13

Figure 6b

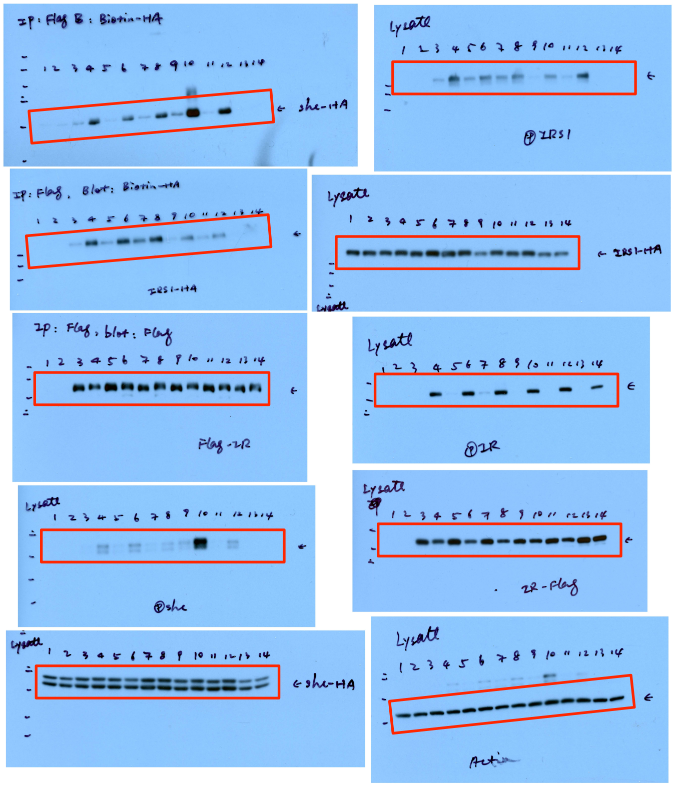

Figure 6c

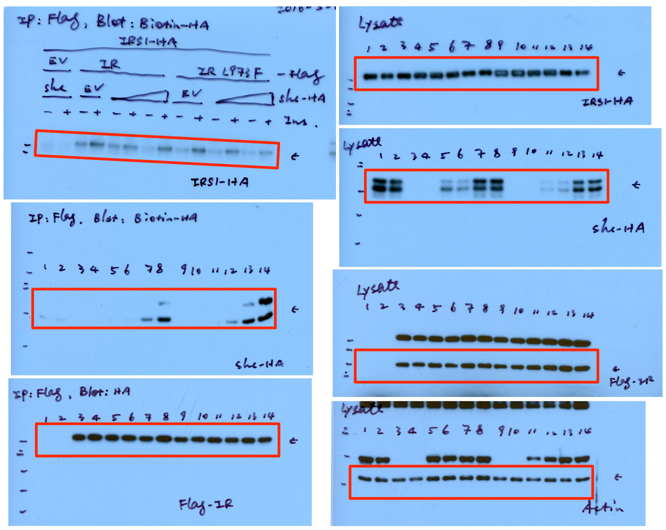

Figure 6d

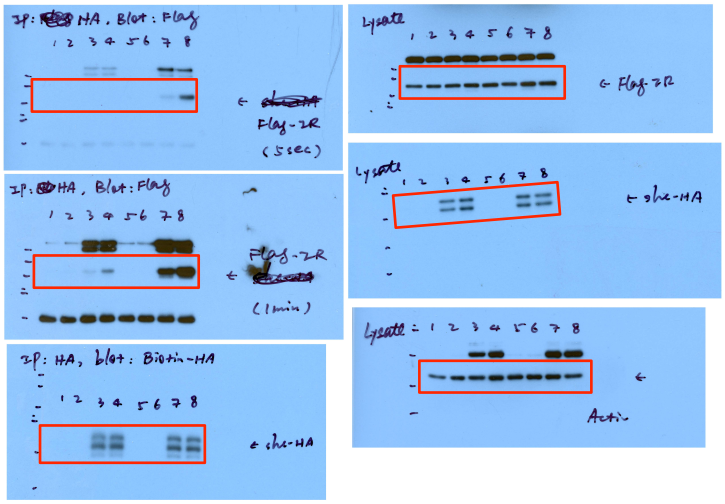

Supplementary Figure 13. Uncut blots. The red sections indicate blot results shown in the indicated figures.

Supplementary Figure 14

Figure 7a

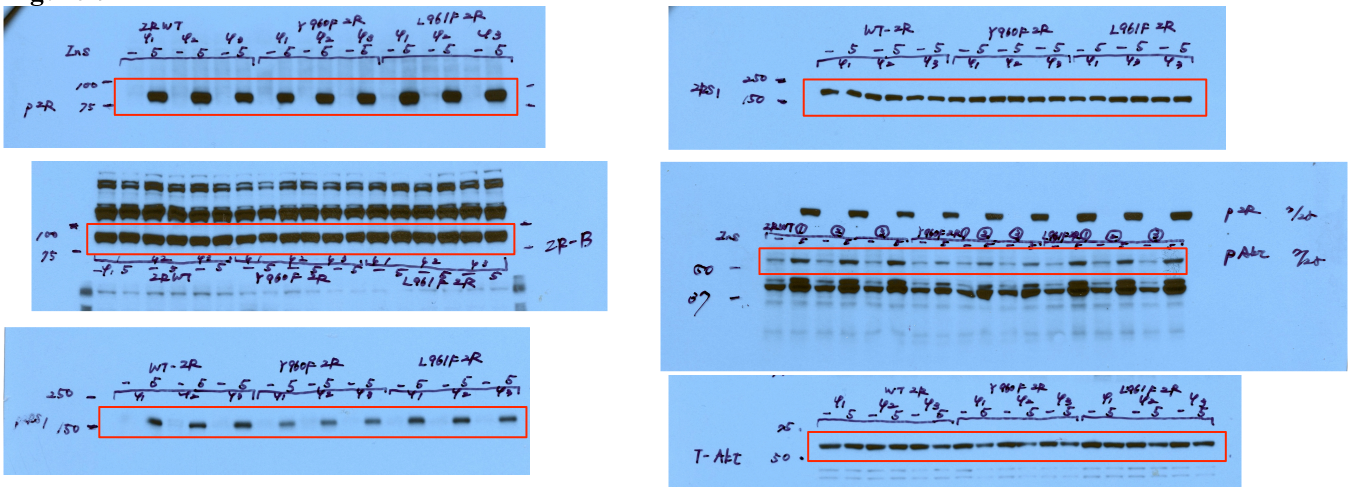

Figure 7e

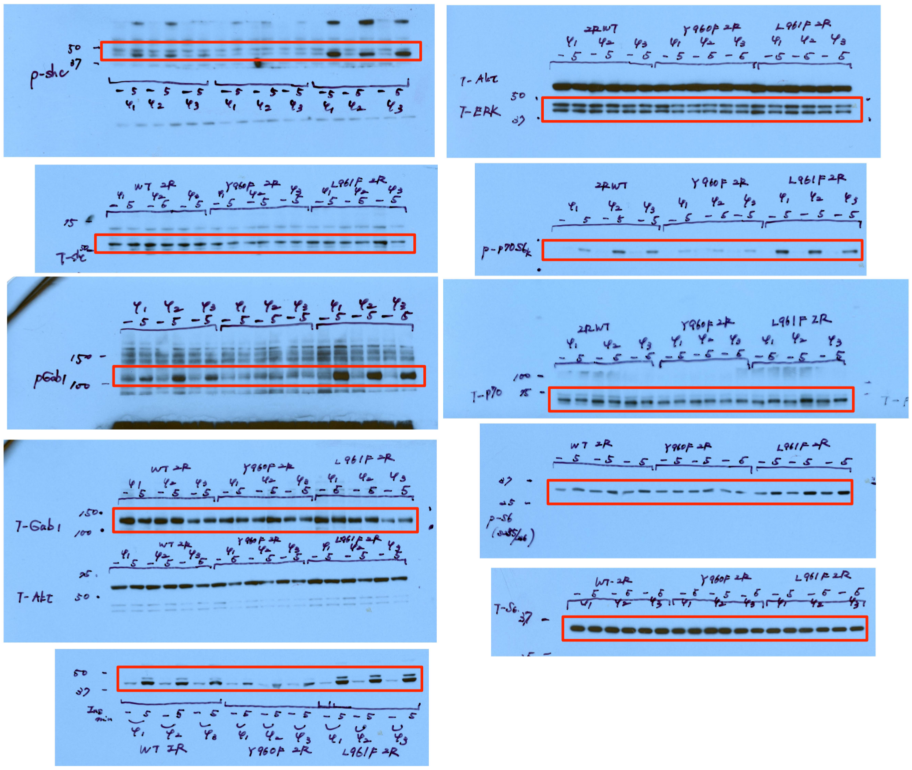

Supplementary Figure 14. Uncut blots. The red sections indicate blot results shown in the indicated figures. Note, the labeling in these films used IR-A amino acid numbers. Thus, Y960F in IR-A is Y972F in IR-B, and L961F in IR-A is L973F in IR-B.

Supplementary Table 1

Top significant genes that were differentially regulated in IR and IGF1R in response to stimulation

Symbol	mRNA Accession	Name	Fold change to basal		Log$_2$ (FC_{IR}/FC_{IGF1R})	p-value	FDR
			IR	IGF1R			
		Group I					
Mcpt8	NM_008572	Mast cell protease 8	1.86	8.63	-2.22	1.71E-05	0.0591
Sema7a	NM_011352	Sema domain, immunoglobulin domain (ig), and GPI membrane anchor 7A	1.12	5.24	-2.22	7.79E-05	0.0976
Dynap	NM_029346	Dynactin associated protein	1.22	4.86	-2.00	2.10E-06	0.0424
Ndst3	NM_031186	N-deacetylase/n-sulfotransferase (heparan glucosaminyl) 3	1.22	4.06	-1.73	9.88E-05	0.112
Hmga2	NM_010441	High mobility group at-hook 2	1.08	3.41	-1.66	6.15E-06	0.0424
Hbegf	NM_010415	Heparin-binding EGF-like growth factor	0.85	2.50	-1.56	0.000217	0.155
Errfi1	NM_133753	ERBB receptor feedback inhibitor 1	1.06	3.07	-1.54	0.000176	0.151
Mir694	NR_030467	microRNA 694	1.03	2.95	-1.52	4.56E-06	0.0424
Tnc	NM_011607	Tenascin C	2.57	7.36	-1.52	4.22E-05	0.0766
Mir763	NR_030434	microRNA 763	0.82	2.28	-1.48	5.56E-05	0.0766
Gm12603	NR_033533	Predicted gene 12603, long non-coding RNA.	1.80	4.59	-1.35	0.000262	0.159
Havcr2	NM_134250	Hepatitis A virus cellular receptor 2	1.23	3.10	-1.33	7.40E-06	0.0437
Mmd	NM_026178	Monocyte to macrophage differentiation-associated	0.93	2.35	-1.33	9.82E-06	0.0451
Dusp4	NM_176933	Dual specificity phosphatase 4	0.91	2.22	-1.29	5.50E-05	0.0766
LOC100503338	XR_106025	PREDICTED: uncharacterized LOC100503338, ncRNA	0.76	1.84	-1.27	2.14E-05	0.0591
Tgfbr1	NM_009370	Transforming growth factor, beta receptor I	0.85	2.03	-1.25	0.000103	0.112
Irs1	NM_010570	Insulin receptor substrate 1	0.66	1.51	-1.18	5.13E-05	0.0766
Arhgap6	NM_001287530	Rho GTPase activating protein 6	0.89	1.88	-1.08	5.07E-06	0.0424
Mgp	NM_008597	Matrix gla protein	0.74	1.56	-1.07	0.000126	0.122
Slco4a1	NM_148933	Solute carrier organic anion transporter family, member 4a1	0.89	1.82	-1.03	0.000111	0.112
D030025P21Rik	NR_028577	RIKEN cDNA D030025P21 gene, long non-coding RNA.	0.66	1.34	-1.02	4.94E-05	0.0766
Socs5	NM_019654	Suppressor of cytokine signaling 5	0.92	1.84	-0.99	0.000196	0.153
Olfr740	NM_146667	Olfactory receptor 740	0.73	1.42	-0.97	0.000245	0.156
Slit2	NM_001291227	Slit homolog 2	0.95	1.85	-0.95	4.70E-05	0.0766
Ccbe1	NM_178793	Collagen and calcium binding EGF domains 1	1.36	2.58	-0.92	7.69E-05	0.0976
Pde11a	NM_001081033	Phosphodiesterase 11A	0.71	1.34	-0.91	0.000147	0.133
Mir133a-2	NR_029901	microRNA 133a-2	0.68	1.20	-0.83	0.000252	0.156
Ngf	NM_013609	Nerve growth factor	1.29	2.25	-0.81	0.000108	0.112
Sestd1	NM_175465	SEC14 and spectrin domains 1	0.84	1.43	-0.78	0.000216	0.155
Mir31	NR_029747	microRNA 31	0.97	1.61	-0.73	0.000253	0.156
Cdc42ep3	NM_026514	CDC42 effector protein (rho GTPase binding) 3	0.78	1.25	-0.69	0.000204	0.154
Klf5	NM_009769	Kruppel-like factor 5	0.81	1.30	-0.68	4.29E-05	0.0766
Fam20c	NM_030565	Family with sequence similarity 20, member C	0.77	1.21	-0.66	0.000281	0.161
Fnip2	NM_001162999	Folliculin interacting protein 2	0.96	1.47	-0.62	0.000228	0.155
Mcl1	NM_008562	Myeloid cell leukemia sequence 1	0.87	1.30	-0.57	0.00018	0.152
		Group II					
Fam43a	NM_177632	Family with sequence similarity 43, member A	0.91	0.51	0.84	0.000137	0.128
Rassf2	NM_175445	Ras association (RalGDS/AF-6) domain family member 2	1.22	0.72	0.76	0.000127	0.122
Snx29	NM_028964	Sorting nexin 29	0.73	0.46	0.65	7.71E-05	0.0976
Gm15716	ENSMUST00000162457	Predicted gene 15716, non-coding RNA	1.15	0.73	0.65	0.000193	0.153
Tapbpl	NM_145391	TAP binding protein-like	0.94	0.60	0.64	0.000285	0.161
Rdh12	NM_030017	Retinol dehydrogenase 12	1.07	0.69	0.63	0.000245	0.156
Cby1	NM_028634	Chibby homolog 1	1.10	0.75	0.55	0.000224	0.155
		Group III					
Stc1	NM_009285	Stanniocalcin 1	4.76	1.21	1.98	0.000205	0.154
Bnip3	NM_009760	BCL2/adenovirus E1B interacting protein 3	3.53	1.22	1.53	2.74E-05	0.0708
Pdk1	NM_172665	Pyruvate dehydrogenase kinase, isoenzyme 1	2.93	1.10	1.41	0.000107	0.112
Fam162a	NM_027342	Family with sequence similarity 162, member A	2.97	1.22	1.29	2.15E-05	0.0591
Pfkl	NM_008826	Phosphofructokinase, liver, b-type	2.60	1.16	1.17	2.08E-05	0.0591
Gys1	NM_030678	Muscle glycogen synthase 1	2.58	1.18	1.13	4.22E-05	0.0766
S1pr1	NM_007901	Sphingosine-1-phosphate receptor 1	1.79	0.89	1.01	0.000104	0.112
Nek6	NM_021606	NIMA (never in mitosis gene a)-related expressed kinase 6	1.74	0.92	0.92	2.12E-05	0.0591
Mir467d	NR_030572	microRNA 467d	1.63	0.87	0.90	0.000279	0.161
Ddit4	NM_029083	DNA-damage-inducible transcript 4	2.53	1.37	0.88	0.000245	0.156
Nos2	NM_010927	Inducible nitric oxide synthase 2	1.90	1.05	0.86	4.32E-05	0.0766
Fbn1	NM_007993	Fibrillin 1	1.52	0.84	0.86	0.000101	0.112
Pgm2	NM_028132	Phosphoglucomutase 2	2.10	1.19	0.83	0.000188	0.152
B430319G15Rik	NR_029474	RIKEN cDNA B430319G15 gene, long non-coding RNA.	1.83	1.04	0.81	0.000185	0.152
Myd88	NM_010851	Myeloid differentiation primary response gene 88	1.37	0.86	0.67	3.89E-05	0.0766
Phospho1	NM_153104	Phosphatase, orphan 1	1.89	1.27	0.58	0.000285	0.161
		Group IV					
Egr1	NM_007913	Early growth response 1	0.22	1.25	-2.50	9.67E-06	0.0451
Egr2	NM_010118	Early growth response 2	0.45	1.18	-1.38	4.17E-05	0.0766

Supplementary Table 2

Selected top differentially regulated pathways by IR and IGF1R from gene set analysis (Reactome)

Highly regulated in IR			Highly regulated in IGF1R		
**Pathway**	**p-value**	**FDR**	**Pathway**	**p-value**	**FDR**
Regulation of Hypoxia inducible factor HIF by oxygen	6.89E-05	0.0214	Integrin cell surface interactions	0.000157	0.0214
Regulation of signaling by Cbl	4.00E-04	0.0253	Membrane binding and targeting of GAG proteins	0.00052	0.0263
Pyruvate metabolism	0.000838	0.0263	Keratan sulfate biosynthesis	0.000967	0.0263
Glycolysis	0.00124	0.0263	Mitochondrial fatty acid beta oxidation	0.00133	0.0263
Glucose metabolism	0.00175	0.0267	Grb2 events in ERBB2 signaling	0.00142	0.0264
TRAF3 dependent IRF activation pathway	0.00181	0.0267	Signaling by Rho GTPases	0.00158	0.0264
Autodegradation of the E3 ubiquitin ligase COP1	0.00187	0.0267	Branched chain amino acid catabolism	0.00163	0.0267
Gluconeogenesis	0.00214	0.0279	Mitotic prometaphase	0.00187	0.0267
Signaling by Wnt	0.00229	0.0279	Golgi associated vesicle biogenesis	0.00207	0.0279
Purine metabolism	0.00279	0.0301	Voltage gated potassium channels	0.00229	0.0279
ER phagosome pathway	0.00465	0.038	Keratan sulfate degradation	0.00544	0.0413
Cholesterol biosynthesis	0.00596	0.0429	Shc related events	0.016	0.0704
Pyrimidine metabolism	0.00736	0.0461	Glycosaminoglycan metabolism	0.0203	0.0802
Diabetes pathway	0.00854	0.0493	Semaphorin interactions	0.0209	0.0809
Bile acid and bile salt metabolism	0.0135	0.0642	Cell surface interactions at the vascular wall	0.0308	0.107

Supplementary Table 3

Top significant genes that were differentially regulated in IR-ECD and IGF1R-ECD in response to stimulation

Symbol	mRNA Accession	Name	Log_2 ($FC_{IR\text{-}ECD}$ /$FC_{IGF1R\text{-}ECD}$)	p-value	FDR
		Group I			
Cck	NM_001284508	Cholecystokinin	0.972	0.00066	0.23
Mmp13	NM_008607	Matrix metallopeptidase 13	0.785	0.000141	0.181
Bnip3	NM_009760	BCL2/adenovirus E1B interacting protein 3	0.774	0.000627	0.23
Hp	NM_017370	Haptoglobin	0.669	0.000523	0.225
Gm25200	ENSMUST00000083094	ncRNA:snoRNA	0.645	0.00028	0.205
Pfkl	ENSMUST00000061829	Unknown protein coding gene	0.587	0.000174	0.181
Cd14	NM_030678	Glycogen synthase 1	0.561	0.00105	0.294
Olfr1507	NM_001170918	Olfactory receptor 1507	0.546	0.000558	0.225
Mir1188	NR_035419	microRNA 1188	0.536	0.00071	0.238
Gm24151	ENSMUST00000082758	ncRNA:snoRNA	0.534	0.000133	0.181
Gm24527	ENSMUST00000082920	ncRNA:snRNA	0.533	0.000802	0.254
Il13ra1	NM_133990	Interleukin 13 receptor, alpha 1	0.529	0.000184	0.181
Ifitm6	NM_001033632	Interferon induced transmembrane protein 6	0.522	0.000282	0.205
Pdc	NM_001159730	Phosducin	0.514	0.00031	0.205
Gm24086	ENSMUST00000175171	ncRNA:snRNA	0.508	0.000498	0.217
Bri3	NM_001163709	Brain protein I3	0.496	0.000242	0.205
Myd88	NM_010851	Myeloid differentiation primary response gene 88	0.487	4.99E-05	0.173
Nek6	NM_021606	NIMA (never in mitosis gene a)-related expressed kinase 6	0.487	0.000357	0.205
Plac8	ENSMUST00000031264	Unknown protein coding gene	0.487	0.000454	0.213
Gp6	NM_001163014	Glycoprotein 6	0.42	0.000223	0.196
		Group II			
Gm10722	NR_028577	Predicted gene, 10722	-1.33	0.000154	0.181
Gm21732	ENSMUST00000179309	Predicted gene, 21732	-1.1	1.08E-06	0.0327
Gm10721	ENSMUST00000143083	Predicted gene, 10721	-1.08	0.000108	0.173
Gm20854	ENSMUST00000181549	Predicted gene, 20854	-0.983	1.70E-05	0.122
Gm20213	ENSMUST00000151376	Predicted gene, 20854, non-coding RNA	-0.958	0.000146	0.181
Olfr1463	NM_001011840	Olfactory receptor 1463	-0.861	0.000214	0.192
LOC102640642	XM_006528646	PREDICTED: uncharacterized LOC102640642, transcript variant X2, mRNA.	-0.825	3.19E-05	0.173
Olfr740	NM_146667	Olfactory receptor 740	-0.814	9.76E-05	0.173
Gm21788	ENSMUST00000177878	Predicted gene, 21788	-0.808	0.000203	0.186
Gm22956	ENSMUST00000122520	Predicted gene, 22956, non-coding RNA	-0.794	0.000101	0.173
Gm20736	NM_001037748	Predicted gene, 20736	-0.786	0.000352	0.205
Astx	XM_006528435	PREDICTED: amplified spermatogenic transcripts X encoded	-0.775	8.74E-05	0.173
Vmn1r114	NM_001166837	Vomeronasal 1 receptor 114	-0.755	0.000107	0.173
5031410I06Rik	NM_207657	RIKEN cDNA 5031410I06 gene	-0.73	8.80E-05	0.173
Gm20815	NM_001017394	Predicted gene, 20815	-0.723	0.000389	0.205
1700091H14Rik	NR_073362	RIKEN cDNA 1700091H14 gene, long non-coding RNA.	-0.701	8.80E-05	0.173
Gm15319	NM_001177408	Predicted gene 15319	-0.699	5.45E-05	0.173
Gm20738	NM_207162	Predicted gene, 20738	-0.692	9.19E-05	0.173
4930433I11Rik	ENSMUST00000171664	RIKEN cDNA 4930433I11 gene	-0.668	0.000202	0.186
D030025P21Rik	XR_382991	RIKEN cDNA D030025P21 gene, long non-coding RNA.	-0.645	0.000194	0.186

Supplementary Table 4

Selected top differentially regulated pathways by IR-ECD and IGF1R-ECD from gene set analysis (Reactome)

Highly regulated in IR-ECD			Highly regulated in IGF1R-ECD		
**Pathway**	**p-value**	**FDR**	**Pathway**	**p-value**	**FDR**
Prolonged ERK activation events	9.87E-06	0.00331	Voltage gated potassium channels	0.00115	0.0215
Regulation of signaling by Cbl	0.000385	0.0215	Keratan sulfate degradation	0.00437	0.0215
Autodegradation of the E3 ubiquitin ligase Cop1	0.000779	0.0215	GPVI mediated activation cascade	0.00437	0.0215
Signaling by Wnt	0.000933	0.0215	ADP signaling through P2PY12	0.0173	0.0473
ER phagosome pathway	0.00108	0.0215	G protein activation	0.0214	0.0553
Triglyceride biosynthesis	0.00152	0.0215	Synthesis of PA	0.0349	0.0831
Metabolism of Vitamins and cofactors	0.00214	0.0215	Formation of fibrin clot clotting cascade	0.0394	0.0904
Pyruvate metabolism	0.00221	0.0215	Inhibition of insulin secretion by adrenaline noradrenaline	0.0394	0.0904
Metabolism of carbohydrates	0.00229	0.0215	Grb2 events in ERBB2 signaling	0.0434	0.0975
Cholesterol biosynthesis	0.00253	0.0215	Acyl chain remodeling of PC	0.0477	0.105

Supplementary Table 5

Top significant genes that were differentially regulated in IR-ICD and IGF1R-ICD in response to stimulation

Symbol	mRNA Accession	Name	Log2 (FC_{IR-ICD} /$FC_{IGF1R-ICD}$)	p-value	FDR
		Group I			
Sema7a	NM_011352	Sema domain, immunoglobulin domain (ig), and GPI membrane anchor 7A	-2.29	5.22E-06	0.0151
Dynap	NM_029346	Dynactin associated protein	-2.23	5.13E-08	0.00212
Mcpt8	NM_008572	Mast cell protease 8	-1.93	4.76E-06	0.0151
Ndst3	NM_031186	N-deacetylase/n-sulfotransferase (heparan glucosaminyl) 3	-1.83	5.26E-06	0.0151
Hmga2	NM_010441	High mobility group at-hook 2	-1.66	4.57E-07	0.00591
Gm12603	NR_033533	Predicted gene 12603, long non-coding RNA.	-1.56	7.29E-06	0.0151
Mir763	NR_030434	microRNA 763	-1.52	3.82E-06	0.0151
Hbegf	NM_010415	Heparin-binding EGF-like growth factor	-1.44	4.05E-05	0.0383
Errfi1	ENSMUST00000073600	ERBB receptor feedback inhibitor 1	-1.43	2.93E-05	0.0319
Tnc	NM_011607	Tenascin C	-1.41	6.91E-06	0.0151
Mir694	NR_030467	microRNA 694	-1.39	7.90E-07	0.00591
Mmd	NM_026178	Monocyte to macrophage differentiation-associated	-1.32	7.87E-07	0.00591
Dusp4	NM_176933	Dual specificity phosphatase 4	-1.32	3.65E-06	0.0151
Havcr2	NM_134250	Hepatitis A virus cellular receptor 2	-1.27	8.58E-07	0.00591
LOC100503338	XR_106025	PREDICTED: uncharacterized LOC100503338, ncRNA	-1.21	2.73E-06	0.0151
Arhgap6	NM_001287530	Rho GTPase activating protein 6	-1.12	2.62E-07	0.00542
Slco4a1	NM_148933	Solute carrier organic anion transporter family, member 4a1	-1.10	5.80E-06	0.0151
Ccnd1	NM_007631	Cyclin D1	-1.08	0.000548	0.0934
Thbd	NM_009378	Thrombomodulin	-1.03	6.27E-06	0.0151
Ccbe1	NM_178793	Collagen and calcium binding EGF domains 1	-1.00	3.12E-06	0.0151
Parvb	NM_133167	Parvin, beta	-0.99	6.09E-06	0.0151
Slc25a37	NM_026331	Solute carrier family 25, member 37	-0.95	1.13E-05	0.0194
Slit2	NM_001291227	Slit homolog 2	-0.83	1.28E-05	0.0203
Hmga1	NM_001025427	High mobility group at-hook1	-0.82	7.07E-06	0.0151
Ngf	NM_013609	Nerve growth factor	-0.78	1.25E-05	0.0203
Fnip2	NM_001162999	Folliculin interacting protein 2	-0.65	1.41E-05	0.0216
Fxyd5	ENSMUST00000079996	FXYD domain-containing ion transport regulator 5	-0.63	2.90E-05	0.0319
Klf5	NM_009769	Kruppel-like factor 5	-0.63	7.85E-06	0.0154
		Group II			
Gm25987	ENSMUST00000083234	Predicted gene 25987, snRNA	0.968	0.000115	0.0522
Hist1h3c	BC120800	Histone cluster 1, h3c	0.911	0.000109	0.0513
3830403N18Rik	NM_027510	RIKEN cdna 3830403n18rik gene	0.884	0.000268	0.0686
Zhx1	NM_001042438	Zinc fingers and homeoboxes 1	0.76	5.71E-05	0.0421
Acox2	NM_001161667	Acyl-coenzyme A oxidase 2, branched chain	0.731	0.000345	0.0762
Fcamr	NM_001170632	Fc receptor, iga, igm, high affinity	0.683	0.00033	0.0762
Ipp	NM_008389	IAP promoted placental gene	0.68	4.08E-05	0.0383
Fig4	NM_133999	FIG4 homolog	0.66	5.35E-05	0.0421
Cdkn2d	NM_009878	Cyclin-dependent kinase inhibitor 2D (p19, inhibits CDK4)	0.654	0.000155	0.0567
H1f0	NM_008197	H1 histone family, member 0	0.65	8.87E-05	0.0489
Gm26425	ENSMUST00000122529	Predicted gene 26425, snRNA	0.649	8.85E-05	0.0489
Hist1h2bg	BC060304	Histone cluster 1, h2bg	0.628	0.000118	0.0522
Pcx	NM_001162946	Pyruvate carboxylase	0.623	0.000267	0.0686
Rassf2	NM_175445	Ras association (ralgds/AF-6) domain family member 2	0.62	6.62E-05	0.0421
Vmn1r129	NM_001166725	Vomeronasal 1 receptor 129	0.588	0.000104	0.0507
		Group III			
Fam162a	NM_027342	Family with sequence similarity 162, member A	0.784	0.000119	0.0522
S1pr1	NM_007901	Sphingosine-1-phosphate receptor 1	0.71	0.000167	0.0591
Serpinb1a	NM_025429	Serine (or cysteine) peptidase inhibitor, clade B, member 1a	0.692	0.000228	0.0647
Ptch1	NM_008957	Patched homolog 1	0.651	0.000233	0.0647
Pfkl	NM_008826	Phosphofructokinase, liver, b-type	0.569	0.000641	0.0985
		Group IV			
Egr1	NM_007913	Early growth response 1	-1.97	6.19E-06	0.0151
Egr2	NM_010118	Early growth response 2	-1.06	3.52E-05	0.0354

Supplementary Table 6

Selected top differentially regulated pathways by IR-ICD and IGF1R-ICD from gene set analysis (Reactome)

Highly regulated in IR-ICD			Highly regulated in IGF1R-ICD		
**Pathway**	**p-value**	**FDR**	**Pathway**	**p-value**	**FDR**
Pyrimidine metabolism	7.82E-06	0.000655	Signaling by Rho GTPases	3.32E-06	0.000445
Oxygen dependent Proline hydroxylation of hypoxia inducible factor alpha	0.000177	0.00439	PPARa activates gene expression	1.87E-05	0.00125
Gluconeogenesis	0.000233	0.00504	Grb2 events in ERBB2 signaling	0.000252	0.00512
Receptor ligand binding initiates the second proteolytic cleavage of Notch receptor	0.000752	0.00742	Branched chain amino acid catabolism	0.000295	0.00564
GPVI mediated activation cascade	0.00194	0.0118	Membrane binding and targetting of GAG proteins	0.000318	0.00592
TIE2 signaling	0.00229	0.0128	Sphingolipid metabolism	0.000331	0.00599
Synthesis secretion and deacylation of Ghrelin	0.0027	0.0138	Golgi associated vesicle biogenesis	0.000344	0.00606
Regulation of Insulin secretion by Glucagon like peptide 1	0.00308	0.0138	Trans Golgi network vesicle budding	0.000432	0.00672
Acyl chain remodeling of PE	0.00437	0.0138	Glycosaminoglycan metabolism	0.000465	0.007
Glycolysis	0.00495	0.0152	Semaphorin interaction	0.000752	0.00742
Synthesis of bile acids and bile salts via 24-Hydroxycholesterol	0.0131	0.0325	Keratan sulfate Keratin metabolism	0.000779	0.00742
Recycling of bile acids and salts	0.0131	0.0325	Mitochondrial fatty acid beta oxidation	0.000808	0.00742
PI3K Akt activation	0.0257	0.0569	Integrin cell surface interactions	0.00111	0.00848
Regulation of hypoxia inducible factor HIF by oxygen	0.0349	0.0739	Mitotic prometaphase	0.00115	0.00859
Phospholipase C mediated cascade	0.0477	0.0966	Cell cycle	0.002	0.0119

Supplementary Table 7

Primer list for quantitative PCR.

Gene Symbols	Primer sequences
Cck_F	GACCCCAGCCATAGAATAAGTG
Cck_R	TTGTTTCCTCATTCCACCTCC
Ccnd1_F	GCCCTCCGTATCTTACTTCAAG
Ccnd1_R	GCGGTCCAGGTAGTTCATG
CD14_F	TTTAACTCTGGCGTAGTCACC
CD14_R	GACCCTCAGAAACCAGGAG
Cdkn2d_F	CTTCATCGGGAGCTGGTG
Cdkn2d_R	AGGCATCTTGGACATTGGG
D030025P21Rik_F	TTCCACCCCAAACTGTCATAG
D030025P21Rik_R	AGTAGCCACCTTACCATTGC
Dynap_F	CTACCACCCTGCCCACATTT
Dynap_R	GGCTTGAGGACCGAGAGTTC
Egr1_F	AGCGCCTTCAATCCTCAAG
Egr1_R	TTTGGCTGGGATAACTCGTC
Egr2_F	TTGACCAGATGAACGGAGTG
Egr2_R	TGCCCATGTAAGTGAAGGTC
Errfi1_F	CCTACAATCTGAACTCCCCTG
Errfi1_R	AAGCTTGACCTTGGAGATGG
Fam162a_F	AAATGCTTCCTCGTCTCTAAGG
Fam162a_R	CTCAAAATCTGTGGGCTTGTG
Fig4_F	ATGGAACGGTGAACTTCTGG
Fig4_R	CTGGCTATTAGGGTCACATACAC
Gm10722_F	TGCCATATTCCATGTCCTACAG
Gm10722_R	TGGCGAGAAAACAGAAAATCAC
Gm21732_F	CATCTGTGTTAGGGTCTCTGTG
Gm21732_R	TGGGCAGGTTGAGAAAGTTC
Hbegf_F	CCCCTATACACATATGACCACAC
Hbegf_R	CAACTTCACTTTCTCTTCACTTTCC
IR_F	AAATGCAGGAACTCTCGGAAGCCT
IR_R	ACCTTCGAGGATTTGGCAGACCTT
IGF1R_F	ATCGCGATTTCTGCGCCAACA
IGF1R_R	TTCTTCTCTTCATCGCCGCAGACT
Il13ra1_F	TGTGGAGTGATTGGAGTGAAG
Il13ra1_R	TGCGACAAAGACTGGAATGG
Mcpt8_F	AAACCTACAACGACTCCATCC
Mcpt8_R	CACAAGCGACAACTGACAATG
Mmp13_F	TTGATGCCATTACCAGTCTCC
Mmp13_R	ACATGGTTGGGAAGTTCTGG
Pfkl_F	GGAAAGCCTATCTCATCCAGC
Pfkl_R	CCATACCCATCTTGCTACTCAG
Rassf2_F	AACCTGCCCTAATCCCATG
Rassf2_R	TAACCCCTCCACAATGAACTC
S1pr1_F	TGTAGACCCAGAGTCCTGC
S1pr1_R	TCAGTTACAGCAAAGCCAGG
Sema7a_F	GTCTACCACTTCAACTTCCCC
Sema7a_R	CCATTACCCCGCCTTTCTAG
Serpinb1a_F	CCCTGTGGATTTTCTGCATG
Serpinb1a_R	GCATTCACCAGCACAAGTTTG
TBP_F	ACCCTTCACCAATGACTCCTATG
TBP_R	TGACTGCAGCAAATCGCTTGG
Tnc_F	ATAGCCAACATCACAGACTCAG
Tnc_R	GCTCGTACTCCACTGTATTTCC
Vmn1r114_F	CCAGTGTCTCCATGGTACTTC
Vmn1r114_R	AGGATAGTTTGGGTTGCTCTG
